# Effects of glycemic control on glucose utilization and mitochondrial respiration during resuscitated murine septic shock

**DOI:** 10.1186/2197-425X-2-19

**Published:** 2014-06-03

**Authors:** Josef A Vogt, Ulrich Wachter, Katja Wagner, Enrico Calzia, Michael Gröger, Sandra Weber, Bettina Stahl, Michael Georgieff, Pierre Asfar, Eric Fontaine, Peter Radermacher, Xavier M Leverve, Florian Wagner

**Affiliations:** Sektion Anästhesiologische Pathophysiologie und Verfahrensentwicklung, Klinik für Anästhesiologie, Universitätsklinikum, Helmhotzstrasse 8-1, Ulm, 89081 Germany; Département de Réanimation Médicale et de Médecine Hyperbare, Centre Hospitalier Universitaire and Laboratoire de Biologie Neurovasculaire et Mitochondriale Intégrée, CNRS UMR 6214 - INSERM U1083, PRES L’UNAM, Angers, 49033 France; Laboratoire de Bioénergétique Fondamentale et Appliquée, Université Joseph Fourier, Grenoble, 38041 France

**Keywords:** Gluconeogenesis, Glucose uptake, Glucose oxidation, Mitochondrial respiration, Apoptosis, AMPK, HO-1

## Abstract

**Background:**

This study aims to test the hypothesis whether lowering glycemia improves mitochondrial function and thereby attenuates apoptotic cell death during resuscitated murine septic shock.

**Methods:**

Immediately and 6 h after cecal ligation and puncture (CLP), mice randomly received either vehicle or the anti-diabetic drug EMD008 (100 μg · g^-1^). At 15 h post CLP, mice were anesthetized, mechanically ventilated, instrumented and rendered normo- or hyperglycemic (target glycemia 100 ± 20 and 180 ± 50 mg · dL^-1^, respectively) by infusing stable, non-radioactive isotope-labeled ^13^C_6_-glucose. Target hemodynamics was achieved by colloid fluid resuscitation and continuous i.v. noradrenaline, and mechanical ventilation was titrated according to blood gases and pulmonary compliance measurements. Gluconeogenesis and glucose oxidation were derived from blood and expiratory glucose and ^13^CO_2_ isotope enrichments, respectively; mathematical modeling allowed analyzing isotope data for glucose uptake as a function of glycemia. Postmortem liver tissue was analyzed for HO-1, AMPK, caspase-3, and Bax (western blotting) expression as well as for mitochondrial respiratory activity (high-resolution respirometry).

**Results:**

Hyperglycemia lowered mitochondrial respiratory capacity; EMD008 treatment was associated with increased mitochondrial respiration. Hyperglycemia decreased AMPK phosphorylation, and EMD008 attenuated both this effect as well as the expression of activated caspase-3 and Bax. During hyperglycemia EMD008 increased HO-1 expression. During hyperglycemia, maximal mitochondrial oxidative phosphorylation rate was directly related to HO-1 expression, while it was unrelated to AMPK activation. According to the mathematical modeling, EMD008 increased the slope of glucose uptake plotted as a function of glycemia.

**Conclusions:**

During resuscitated, polymicrobial, murine septic shock, glycemic control either by reducing glucose infusion rates or EMD008 improved glucose uptake and thereby liver tissue mitochondrial respiratory activity. EMD008 effects were more pronounced during hyperglycemia and coincided with attenuated markers of apoptosis. The effects of glucose control were at least in part due to the up-regulation of HO-1 and activation of AMPK.

**Electronic supplementary material:**

The online version of this article (doi:10.1186/2197-425X-2-19) contains supplementary material, which is available to authorized users.

## Background

Hyperglycemia is the hallmark of sepsis-induced metabolic stress [1, 2] and is caused by both inhibition of insulin-mediated glucose uptake [2], i.e., insulin resistance [1–3], insufficient insulin secretion [4], as well as increased hepatic glucose production from glycogenolysis and gluconeogenesis [2]. It is well-established that sepsis-induced hyperglycemia is associated with oxidative stress [5, 6], which causes mitochondrial dysfunction and organ damage in the kidney [7, 8], the heart [8], and the liver [8] when present over prolonged time. Maintaining normoglycemia by insulin control at different nutritional intake levels protected against such mitochondrial and organ damage [7, 8]. These studies indicated that glycemia-independent effects of insulin appeared to be marginal in this context: blood glucose control with insulin did not enhance the protective effect of normoglycemia *per se*, and combining hyperglycemia and hyperinsulinemia was associated with the most pronounced mitochondrial damage and organ injury [8]. Moreover, insulin has marked immunologic effects [2], e.g., by inhibition of glycogen synthase kinase-3β [9, 10]. Finally, tight blood glucose control with insulin may lead to episodes of deleterious hypoglycemia [11, 12]. Metformin, a first line-defense drug for the treatment of type 2 diabetes may theoretically circumvent the undesired side effects of insulin [13]. Metformin attenuated both the LPS-induced hyper-inflammatory response after partial hepatectomy [14] and oxidative stress due to mild inhibition of the mitochondrial complex I [15], decreases hepatic gluconeogenesis [16], and improves whole-body glucose oxidation during hyperglycemia [17]. Metformin also stabilizes mitochondrial functioning by reducing the transition pore opening, which protects against ischemia- [18], oxidant- [19, 20], or hyperglycemia-induced [21] cell death. Finally, it stimulates mitochondrial biogenesis via PG1α signaling [22], which in turn coincided with survival in patients with sepsis [23]. However, metformin use is limited by its rare but serious side effect, lactic acidosis, which could develop in this context due to the frequent renal impairment associated with septic shock [24–26]. Since we recently demonstrated acute kidney injury during murine septic shock [27], we therefore tested the hypothesis whether lowering glycemia using EMD008 would allow improving mitochondrial function and thereby attenuate apoptotic cell death. EMD008 was chosen because it lowers ATP/ADP ratios similar to metformin, which should activate AMPK and thereby improve hepatic and skeletal muscle glucose utilization [28] and uptake. EMD008 alleviates glucotoxic stimulation of apoptosis without or induction of hypoglycemia. In contrast to metformin, these effects are not based on an inhibition of mitochondrial complex I and the reduction of mitochondrial respiration and cytosolic NADH/NAD^+^ ratio is less pronounced, reducing the risk of lactic acidosis (all findings pertaining to EMD008 are personal communications of XL). Septic shock with normotensive, hyperdynamic hemodynamics resulting from fluid resuscitation and continuous i.v. noradrenaline was investigated in order to exclude any systemic hemodynamic effect on metabolism: in fact, the above-mentioned data originate from long-term experiments in awake, spontaneous breathing animals presenting with lower-organ O_2_ supply than in healthy control animals [7, 8].

## Methods

### Anesthesia, surgical instrumentation, and experimental protocol

The study protocol was approved by the University Animal Care Committee and the federal authorities for animal research of the Regierungspräsidium Tübingen, Baden-Württemberg. Male C57BL/6 J mice (body weight 23 to 29 g, age 10 to 16 weeks) were used for the experiments, which were performed in adherence to the National Institutes of Health Guidelines on the Use of Laboratory Animals. The animals, which did not undergo mechanical ventilation and surgical instrumentation, served as controls for tissue immunoblotting and electrophoretic mobility shift assay (EMSA). The anesthesia, cecal ligation and puncture (CLP) procedure and the surgical instrumentation have been described in detail previously [27, 29]. Mice were anesthetized with sevoflurane and received s.c. buprenorphine together with acetated Ringer's solution containing glucose (4 or 12 mg · g^-1^ for normoglycemic and hyperglycemic animals). A midline laparotomy was performed to identify and ligate the cecum followed by a single puncture (18-gauge needle). After squeezing to expel stool, the cecum was returned into the abdominal cavity. Postoperatively, water and food were provided *ad libitum*. After 6 h, mice received a second s.c. injection including buprenorphine, acetated Ringer's solution containing glucose as described above together with ceftriaxone and clindamycin (each with 30 μg · g^-1^). Fifteen hours post CLP, mice were anesthetized with sevoflurane followed by i.p. ketamine (120 μg · g^-1^), midazolam (125 μg · g^-1^), and fentanyl (0.25 μg · g^-1^). After placement of the animal on the procedure bench equipped with a heating pad and a lamp, a rectal temperature probe was inserted. The anterior neck was incised to expose the trachea, the right internal jugular vein, and the right carotid artery. The trachea was intubated, and the lungs were mechanically ventilated with a pressure-controlled, lung-protective ventilation strategy using a small animal ventilator (FlexiVentTM, Scireq®, Montreal, Canada) [27, 29, 30]. After a lung recruitment maneuver, respirator settings were FiO_2_ 0.5, tidal volume 6 to 8 μL · g^-1^ (titrated to maintain arterial PCO_2_ at 30 to 40 mmHg), respiratory rate 160 breaths · min^-1^, inspiratory/expiratory time ratio 1:2, and PEEP 5 cm H_2_O. Catheters were inserted into the jugular vein, the carotid artery, and the bladder. Anesthesia was maintained with continuous i.v. ketamine, fentanyl and midazolam, titrated to reach deep sedation and analgesia as documented by complete tolerance against noxious stimuli. Normotensive hemodynamics (i.e., mean arterial pressure > 55 mmHg) were achieved by i.v. hydroxyethyl starch (maximum infusion rate 20 μL · g^-1^ · h^-1^) in a balanced electrolyte solution (Tetraspan 6%, Braun, Melsungen, Germany), and, if needed, together with continuous i.v. norepinephrine. Animals were randomly assigned to injection of vehicle (0.9% saline) or EMD008 (100 μg · g^-1^) twice s.c., immediately and 6 h after the CLP procedure and i.v. after insertion of the jugular vein catheter. In addition, animals received either 1 or 2 mg · g^-1^ · h^-1^ continuous i.v. glucose to achieve a normo- (target glycemia 100 ± 20 mg · dL^-1^; vehicle *n* = 8, EMD008 *n* = 7) or hyperglycemic (target glycemia 180 ± 50 mg · dL^-1^; vehicle *n* = 10, EMD008 *n* = 11) conditions, respectively. Infused glucose 50% was given as stable, non-radioactive-labeled 1,2,3,4,5,6-^13^C_6_-glucose. After 5 h, the animals were killed through blood withdrawal via the vena cava inferior.

### Cell extracts, immunoblots, and comet assay

Immediately postmortem, the liver was removed, snap-frozen, and stored at -80°C. Frozen tissue was homogenized and lysed in lysing buffer 100 mM Tris pH 7.5; 500 mM NaCl; 6 mM EDTA; 6 mM EGTA; 2% Triton-X-100; 1% NP 40; 20% glycerol; protease inhibitors (β-glycerolphosphat 2 mM; DTT 4 mM; leupeptin 20 μM; pNPP (p-nitrophenylphosphate) 4 mM; natriumorthovanadate 0.2 mM). To assess the expression of AMPK-α, p(Thr172)-AMPK-α, activated caspase-3, and heme oxygenase-1 (HO-1) equal total protein aliquots (20 μg) were separated by SDS-PAGE and transferred by western blotting. After blocking, the gel membranes were incubated with primary antibodies (anti-AMPK-α; anti-p(Thr172)-AMPK- α; anti-Bax; anti-cleaved caspase-3 (Cell Signaling, Danvers, MA, USA); anti-HO-1 (Abcam, Cambridge, NY, USA)). Actin, with a primary antibody from Santa Cruz, Dallas, TX, USA, was used as loading control. The primary antibodies were detected using horseradish peroxidase-conjugated secondary antibodies (Cell Signaling, Danvers, MA, USA or Santa Cruz, Dallas, TX, USA). The membranes were subjected to chemiluminescence using SuperSignal West Femto Maximum Sensitivity Substrate (Thermo Fisher Scientific, Waltham, MA, USA). Exposed films were scanned, and intensity of immunoreactivity was measured using NIH ImageJ software (http://rsb.info.nih.gov/nih-image). Protein expressions are presented as *x*-fold increase over control values. These control values were taken from samples of two healthy, native animals, which were co-separated on each gel. AMPK stimulation is expressed as the ratio of p(Thr172)-AMPK-α/AMPK-α.

Single-cell gel electrophoresis allowed assessing the oxidative deoxyribonucleic acid (DNA) damage (‘tail moment’ in the alkaline version of the comet assay) [27, 31]. Immediate postmortem biopsies were placed in buffer containing Na-ethylenediaminetetraacetic acid and minced to obtain a cell suspension. Two agarose gel slides were prepared from each biopsy. The mean tail moment of 100 nuclei analyzed per slide was used for each animal.

### Glucose metabolism

For the measurement of blood glucose concentrations and ^13^C_6_-glucose tracer enrichment, plasma samples were spiked with 6,6-^2^H_2_-glucose for concentration determination, with an amount targeted to achieve a ^2^H_2_-tracer mole fraction of 50%. The spiked samples were derivatized with *N*-methyl-bis(trifluoroacetamide) (MBTFA, abcr, Karlsruhe, Germany) to obtain the trifluoroacetyl-glucose derivative [31]. The latter was analyzed by GC/MS under electron impact determination, and the signals at (m/z) 319, 321, and 325 were recorded for the ^2^H_2_- and ^13^C_6_-tracer mole fraction determination. Expiratory gas, 1 ml, was continuously collected as an aliquot of 60 to 80 respiratory cycles from the expiratory branch to determine both expiratory CO_2_ concentration and ^13^CO_2_ tracer enrichment using GC/MS and measuring the masses m/z 44 and m/z 45. CO_2_ production rates were calculated as the product of tidal volume, respiratory rate, and CO_2_ concentration. During steady-state conditions, the glucose rate of appearance (Ra) is derived from the arterial plasma isotope enrichment (atom percentage excess or APE) according to Equation 1:
1

where APE_pl_ is the isotope enrichment in the plasma, and *F* is the infusion rate of the labeled glucose [32]. Endogenous glucose production is the difference between Ra and the total exogenous glucose infusion, 2 · F. Since glycogenolysis most likely was hardly present any longer [33], endogenous glucose production is assumed to equal gluconeogenesis (*GNG*).

### Mitochondrial respiration

The activity of the mitochondrial respiratory chain was measured in immediate postmortem homogenized liver samples with disrupted cell membranes and intact mitochondria using ‘high-resolution respirometry’ (Oxygraph-2 k respirometer; OROBOROS Instruments Corp, Innsbruck, Austria) [31, 34]. Data reported are maximal oxidative phosphorylation, i.e., O_2_ consumption after addition of substrates of complexes I and II (pyruvate 10 mM, malate 5 mM + glutamate 10 mM, and succinate 10 mM) and ADP (5 mM) and maximal O_2_ consumption in the uncoupled state, i.e., after 4-(trifluoromethoxy) phenylhydrazone (FCCP), respectively.

### Tracer data evaluation

The 1,2,3,4,5,6-^13^C_6_-glucose plasma and mixed expiratory ^13^CO_2_ tracer isotope enrichment data allowed for calculating the flow rates of gluconeogenesis and glucose oxidation. During steady state conditions, whole-body glucose uptake (*uptake*) equals the sum of gluconeogenesis (GNG) and exogenous glucose infusion. In order to determine the relation between glucose uptake and glycemia as well as a possible effect of EMD008, the measured flux data were analyzed using a mathematical model based on the assumption that these flow rates are directly closely linked to the plasma glucose availability (*conc*) and the AMPK activation (*ampk*). The latter was integrated into that model because AMPK is a crucial regulator of cellular glucose disposal [28] that also contributes to the glycemic effect of metformin [35]. A potential link between flow rates and the two controlling factors, glycemia and AMPK activation, was explored by regression analysis. Since all the variables involved in the regression are determined with a certain measurement error, an *error in all variables* approach was used adjusting all measured flow rates and controlling factors as close as possible to their corresponding measurement values, satisfying the following Equation 2:
2

where the coefficients *k*_1_ and *k*_2_ quantify the impact of the glucose concentration and AMPK activation, respectively. In analogy, an equation was defined to express gluconeogenesis as a function of the controlling factors. An impact is established if the regression estimate for the corresponding coefficient is significantly different from 0. Hyperglycemic and normoglycemic animals were analyzed with the same set of coefficients, assuming that the sensitivity of flow rates to changes in the control processes remains the same for all conditions. Vehicle and EMD008-treated animals were analyzed with both distinct *and* the same sets of coefficients to explore whether AMPK activity alone is sufficient to explain the EMD008 effect. Details of the regression approach are given in the Additional file 1.

### Statistical analysis

Differences between groups were analyzed with a one-way Kruskal-Wallis analysis of variance on ranks followed by a *post hoc* Dunn's test. Differences between EMD008 and vehicle were tested with a student's *t* test or a Mann-Whitney rank sum as appropriate according to data distribution. The regression was performed using the STAN software package [36], which expands on the Bayesian statistical package WinBUGS [37] (BUGS = Bayesian statistics using Gibbs sampling). It allows a flexible definition of the statistical model and makes no assumption about the distribution of a test statistics but estimates it using MCMC sampling given the data and eventually prior information. It thereby provides reliable estimates of the 95% confidence range for parameters of interest, which are used here as indicator for significance.

## Results

Despite the maximum colloid infusion rate allowed by the protocol, all mice needed continuous i.v. norepinephrine to maintain target hemodynamics, which were lower in the normoglycemic EMD008-treated animals (0.009 (0.08 to 0.012) μg · g^-1^ · h^-1^) vs. all other groups (*p* < 0.05, hyperglycemia, vehicle 0.025 (0.011 to 0.062), hyperglycemia EMD008 0.030 (0.022 to 0.183), normoglycemia vehicle 0.031 (0.027 to 0.205)). Table 1 summarizes the parameters of systemic and regional macro- and microcirculatory perfusion, gas exchange, and acid-base status, without inter-group difference. However, in the normoglycemia group, EMD008 treatment prevented the progressive fall of arterial pH present in the other experimental groups. Table 2 summarizes the results of the metabolic measurements. According to the protocol, blood glucose levels were higher in the hyperglycemia groups, which coincided with more pronounced hyperlactatemia. EMD008 lowered blood glucose concentrations in these animals, whereas it had no effect in the normoglycemic mice. While whole-body CO_2_ production and direct aerobic glucose oxidation were comparable between groups, hepatic gluconeogenesis was significantly lower in the hyperglycemic animals. In the latter, EMD008 further increased gluconeogenesis. Table 2 also demonstrates that hyperglycemia lowered both maximal oxidative phosphorylation and maximal electron transfer capacity in the uncoupled state in liver mitochondria and that EMD008 treatment was associated with increased mitochondrial respiration, no matter what the glycemia level is. Table 3 summarizes the immune biology results. Hyperglycemia was associated with decreased phosphorylation of AMPK, and EMD008 attenuated this effect. EMD008 significantly attenuated the expression of Bax during hyperglycemia and that of activated caspase-3 under normoglycemia. While EMD008 increased HO-1 expression during hyperglycemia, it had no effect in the normoglycemic animals. Liver tissue tail moment obtained from the comet assay was comparable in all groups.

**Table 1:** **Parameters of macro- and microcirculatory hemodynamics, blood gases, and acid-base status**

		Hyperglycemia		Normoglycemia	
		Vehicle	EMD008	Vehicle	EMD008
Heart rate (beats · min^-1^)	Start	422 (375;510)	446 (379;481)	459 (406;509)	351 (365;392)
	End	452 (420;489)	437 (390;456)	495 (478;505)	352 (350;384)
Mean arterial pressure (mmHg)	Start	62 (60;67)	65 (60;67)	61 (59;67)	71 (64;74)
	End	60 (57;64)	58 (53;64)	62 (58;65)	67 (65;75)
Portal venous flow (mL · min^-1^)	Start	5.2 (4.3;8.0)	7.2 (5.6;7.8)	9.4 (7.9;11.6)	5.8 (4.5;7.0)
	End	5.1 (4.0;6.1)	6.2 (5.4;6.8)	8.9 (8.0;11.8)	5.3 (4.2;6.5)
Liver μ-vascular flow (AU)	Start	132 (121;142)	136 (121;160)	135 (118;180)	140 (124;159)
	End	124 (105;141)	118 (111;130)	106 (87;115)	111 (101;124)
Liver μ-Hb O_2_ saturation (%)	Start	65 (63;67)	71 (66;73)	67 (63;72)	66 (63;74)
	End	65 (63;69)	67 (64;71)	64 (59;65)	67 (62;71)
Arterial PO_2_ (mmHg)	Start	336 (311;363)	350 (345;364)	368 (360;379)	347 (340;354)
	End	314 (277;-334)	344 (324;350)	312 (301;322)	328 (323;340)
Arterial PCO_2_ (mmHg)	Start	34 (26;35)	30 (29;33)	28 (27;30)	31 (28;33)
	End	35 (32;38)	40 (35;43)	29 (28;31)	30 (29;34)
Arterial pH	Start	7.34 (7.31;7.36)	7.32 (7.29;7.36)	7.36 (7.31;7.40)	7.39 (7.33;7.40)
	End	7.31 (7.26;7.33)$	7.20 (7.02;7.30)$	7.30 (7.25;7.33)$	7.40 (7.32;7.41)
Arterial base excess (mmol · L^-1^)	Start	-9.2 (-11.0;-6.3)	-9.2 (-9.6;-8.4)	-10.0 (-10.3;-7.0)	-7.2 (-9.3;-7.0)
	End	-9.3 (-10.5;-4.6)	-13.1 (-17.2;-9.3)$	-11.4 (-12.6;-9.5)$	-7.6 (-9.1;-6.4)

Liver μ-vascular flow and μ-Hb O_2_ saturation are capillary blood flow and hemoglobin O_2_ saturation, respectively. All data are median (quartiles); $*p* < 0.05 start vs. end within one group.

**Table 2:** **Parameters of glucose metabolism and mitochondrial respiratory activity**

	Hyperglycemia		Normoglycemia	
	Vehicle	EMD008	Vehicle	EMD008
Arterial glucose (mg · dL^-1^)	151 (146;202)#	138 (128;142)#§	104 (94;120)	99 (71;122)
Arterial lactate (mmol · L^-1^)	3.2 (2.6;3.5)#	3.8 (3.0;4.8)#	2.1 (1.9;2.5)	1.8 (1.5;1.9)
CO_2_ production (μL · min^-1^)	27 (25;32)	29 (27;31)	27 (25;27)	24 (22;27)
Gluconeogenesis (mg · g^-1^ · h^-1^)	0.31 (0.26;0.35)#	0.38 (0.33;0.40)#§	0.40 (0.38;0.45)	0.53 (0.49;0.53)**
Glucose oxidation (% isotope infusion)	63 (57;67)	62 (59;64)	62 (60;64)	63 (59;66)
JO_2_-OXPHOS (pmol · s^-1^)	116 (97;122)#	136 (134;160)#§	150 (136;177)	185 (167–197)§
JO_2_-ETC (pmol · s^-1^)	147 (130;159)#	166 (154;194)#*	183 (171;193)	210 (203;238)§

JO_2_-OXPHOS and JO_2_-ETC are maximal oxidative phosphorylation at optimal substrate availability and maximal electron transfer capacity in the uncoupled state, respectively, as O_2_ consumption rate per 10^6^ cells. All data are median (quartiles). #*p* < 0.05 vs. normoglycemia; §*p* < 0.05 vs. vehicle; *p = 0.064 vs. vehicle; **p = 0.073 vs. vehicle.

**Table 3:** **Signal transduction and mediator proteins**

	Hyperglycemia		Normoglycemia	
	Vehicle	EMD008	Vehicle	EMD008
Tail moment	0.5 (0.5;0.6)	0.6 (0.5;0.7)	0.5 (0.4;0.6)	0.6 (0.4;0.7)
HO-1	1.6 (1.6;1.7)	2.2 (2.0;2.3)§	1.7 (1.6;1.8)	1.9 (1.6;2.2)
Bax	1.7 (1.5;1.9)#	1.3 (1.3;1.3)§	1.3 (1.1;1.3)	1.3 (1.3;1.4)
Caspase-3	1.1 (1.1;1.2)	1.0 (0.9;1.1)	1.1 (1.0;1.3)	0.7 (0.7;0.8)§
AMPK activation	0.6 (0.6-0.7)#	0.8 (0.8;0.9)§	0.8 (0.8;0.9)	0.8 (0.7;0.8)
*Total AMPK*	1.0 (0.99;1.05)	1.03 (0.98;1.08)	1.0 (0.98;1.02)	1.06 (0.99-1.08)

AMPK activation is expressed as TH172-phosphorylated AMPK in percentage of total AMPK. All values are expressed as fold over control values from animals that had not undergone surgery. All data are median (quartiles). #*p* < 0.05 vs. normoglycemia; §*p* < 0.05 vs. vehicle.

**Figure 1:**
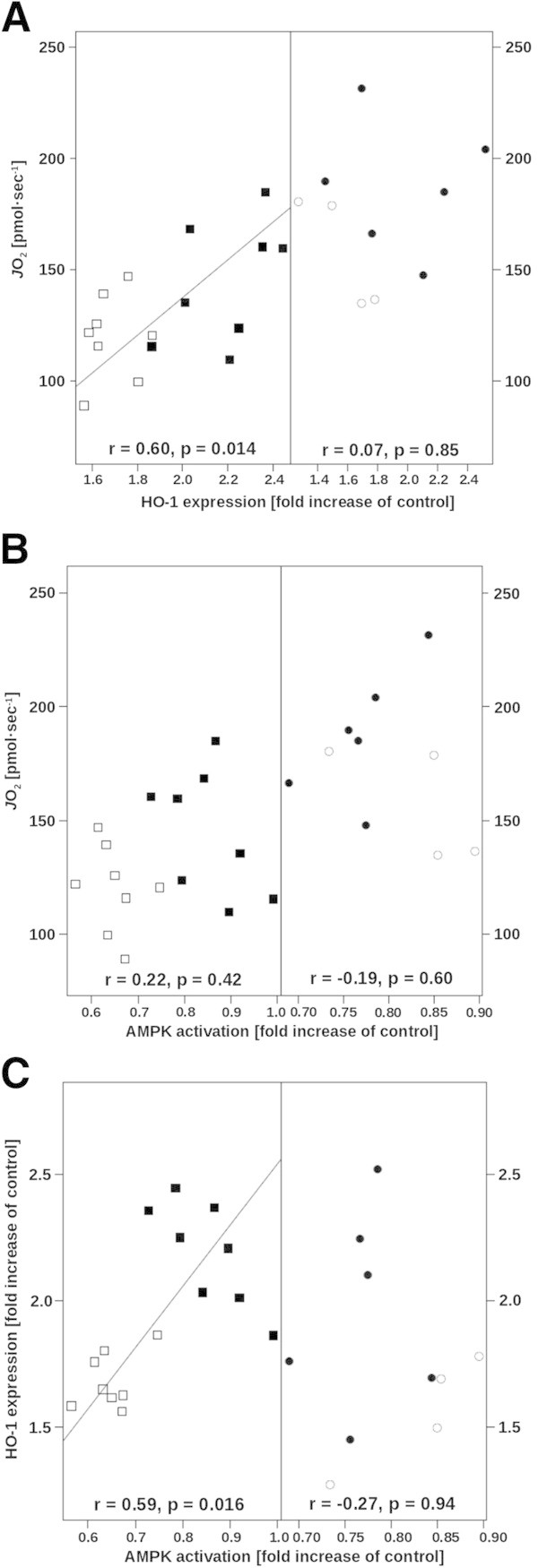
**Relationships between maximal mitochondrial oxidative phosphorylation, HO-1 expression, and AMPK activation.** Maximal oxidative phosphorylation plotted as a function of HO-1 expression **(A)** and of AMPK activation **(B)**, and HO-1 expression plotted as a function of AMPK activation **(C)**. Hyperglycemic animals (squares) are shown on the left, normoglycemic mice (circles) on the right panels each; vehicle-treated mice are represented by open symbols and EMD008-treated animals by black symbols. Overall correlation between mitochondrial oxidative phosphorylation, HO-1 expression, and AMPK activation was r = 0.15 (p = 0.47) and r = 0.2 (p = 0.31), respectively. Overall correlation between HO-1 expression and AMPK activation was r = 0.33 (p = 0.098). Due to technical difficulties, the number of observations was reduced in the normoglycemic groups, which limits the statistical reliability of the evaluation.

**Figure 2:**
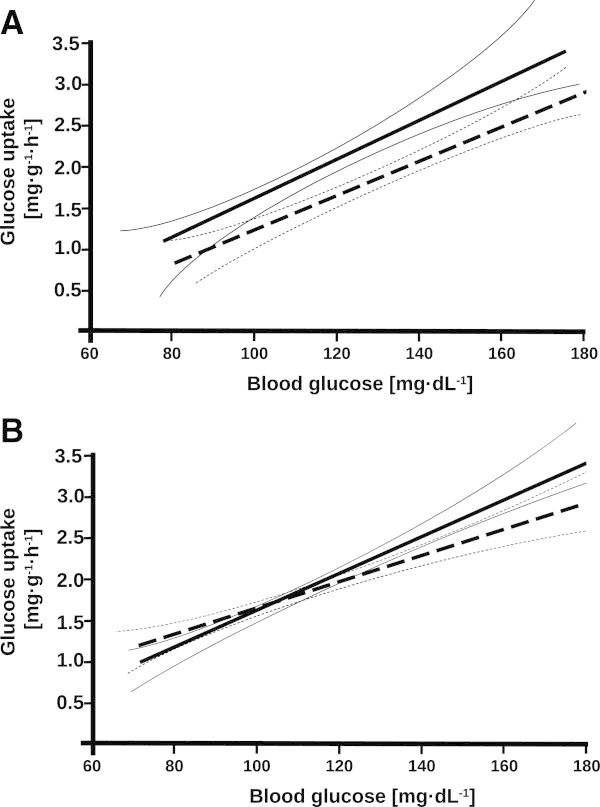
**Results of mathematical modeling of isotope enrichment data presented as glucose uptake.** Lines represent mean and 95% confidence intervals for EMD008- (straight lines) and vehicle-treated (broken lines) animals. There is a loose correlation between AMPK activation values and glycemia and hence some of the effects of AMPK activation can be carried over to the glucose variable, when the impact of AMPK activation is ignored in Equation 2. **(A)** Regression lines obtained under these conditions and when different parameters were used for the EMD008 and for control groups. At comparable glucose concentrations, the predicted uptake is higher for the EMD008 group. When the AMPK effect is considered and one set of coefficients is used for all groups, then a significant effect of AMPK on glucose uptake can be established. Since a dependency on two factors is difficult to visualize, the actual values for AMPK used in the term *k*_*2*_ *AMPK* in Equation 2 are expressed by a glycemia-dependent term derived from a linear approximate relation between AMPK activation and glycemia, which allows to express glucose uptake as a function of glycemia alone. **(B)** An approximate replacement which demonstrates that EMD008 increased the slope of the relation between glucose disposal and glycemia [28].

Figure 1 shows the relation between mitochondrial respiration and HO-1 expression or AMPK activation. During hyperglycemia, the maximal mitochondrial oxidative phosphorylation rate was directly related to HO-1 expression (*p* < 0.05) (Figure 1A, left panel), while there was no relationship during normoglycemia (Figure 1A, right panel). In contrast, mitochondrial oxidative phosphorylation was unrelated to AMPK activation, no matter what the glycemia level is (Figure 1B). During hyperglycemia, HO-1 expression was directly related to AMPK activation (*p* < 0.05) (Figure 1C, left panel), while no significant relation was present during normoglycemia (Figure 1C, right panel). Figure 2 summarizes the results of the mathematical modeling of the isotope enrichment data. Assuming in Equation 1 that AMPK activation was not affected by the level of glycemia, the mathematical modeling demonstrated that EMD008 caused an upward shift of the linear relationship between glucose disposal and blood glucose concentration without affecting its slope, i.e., EMD008 treatment increased cellular glucose uptake, no matter what the actual level of glycemia is (Figure 2A). In turn, when the effect of glycemia on AMPK activation was taken into account in Equation 1, EMD008 increased the slope of the relation between glucose disposal and glycemia (Figure 2B), i.e., increased glucose disposal at a given level of glycemia.

## Discussion

This study was to test the hypothesis whether glycemic control using the newly developed anti-diabetic drug EMD008 would improve glucose uptake and thereby increase the mitochondrial respiratory activity of murine resuscitated, polymicrobial septic shock. EMD008 was chosen to reduce blood sugar levels rather than insulin or metformin to avoid any insulin-induced hypoglycemia and lactic acidosis, respectively. The major findings were as follows: (i) EMD008 increased both liver tissue oxidative phosphorylation and maximal O_2_ uptake, (ii) this effect was even more pronounced during normoglycemia, (iii) associated with attenuated markers of tissue apoptosis, and (iv) associated with an up-regulation of HO-1 and activation of AMPK.

Hyperglycemia was associated with significantly lower mitochondrial oxidative phosphorylation and maximal O_2_ consumption in the uncoupled state. Controlling glycemia restored mitochondrial capacity, and EMD008 further enhanced this effect. Our findings of a hyperglycemia-induced depression of mitochondrial capacity extends previous reports on tissue respiration by Vanhorebeek in a long-term, un-resuscitated rabbit model of burn injury-induced critical illness [7, 8], inasmuch as hyperglycemia has similar effects during the acute phase of noradrenaline-resuscitated murine septic shock. At a first glance, increased tissue respiration could be induced by the noradrenaline intervention alone as reported by other authors for liver mitochondrial respiration during long-term porcine endotoxemia [38]. These authors, however, compared noradrenaline treatment to fluid resuscitation alone, whereas in our experiment, all mice received continuous i.v. noradrenaline to achieve target hemodynamics. Furthermore, we explicitly studied the effect of hyperglycemia in comparison to normoglycemia, while glycemia was maintained between 63 and 108 mgmol · dL^-1^ in that study.

Hyperglycemia *per se* was associated with significantly higher Bax expression, while EMD008 attenuated caspase-3 activation during normoglycemia. As a whole, these findings agree with various previous reports that hyperglycemia causes liver tissue apoptosis [39–42], which can be prevented by anti-diabetic drugs such as metformin [20, 21, 43], even during acute stress states [44]. Attenuation of apoptosis resulting from glucose control *per se*[39–42] and/or metformin treatment [20, 21, 43, 44] is mainly referred to decreased oxidative stress associated with restoration of normoglycemia [7, 8]. Metformin reduces oxidative stress due to inhibition of complex I and a consecutive decrease of mitochondrial O_2_ uptake [15, 19, 21, 45]. EMD008 treatment coincided with increased mitochondrial respiration, no matter what the level of glycemia is. Hence, it exhibited protective effects on mitochondrial function without reducing mitochondrial O_2_ consumption. Moreover, the tail moment in the comet assay, which previously proved to be a sensitive marker of tissue oxidative stress both in our murine model of resuscitated septic shock [31, 46] as well as in hyperglycemic rats [40, 42], did not show any inter-group difference. Consequently, a postulated key mechanism of metformin action, namely, complex I inhibition that reduces both ROS production and mitochondrial O_2_ consumption, may not contribute to EMD008 effects and other mechanisms are likely to be involved. Figure 1 demonstrates that at each level of glycemia, EMD008 treatment coincided with the highest individual values of HO-1 expression. During hyperglycemia, EMD008 not only significantly increased HO-1 expression when compared to the vehicle-treated animals, but there also was a significant direct relation between mitochondrial respiratory capacity and HO-1 expression. Up-regulation of HO-1 is an adaptive response against oxidative stress-induced mitochondrial dysfunction during streptozotocin-induced diabetes [47]. During the transition from acute to chronic 3,5-diethoxycarbonyl-1,4-dihydrocollidine hepatotoxicity [48], it counteracts mitochondrial dysfunction and energetic failure. Induction of HO-1 induction was associated with increased release of anti-inflammatory cytokines and hepatic mitochondrial biogenesis in endotoxic shock [49], which ultimately protected against otherwise lethal *Staphylococcus aureus* sepsis [50]. Finally, activation of HO-1 with hemin improved glucose metabolism in diabetic rats [51] as a result of synergistic interaction between HO-1 and AMPK [52]. In our experiments, during hyperglycemia, HO-1 expression was directly related to AMPK activation, and the mathematical modeling of the glucose enrichment data depicted in Figure 2 suggests that the EMD008-associated increase in cellular glucose utilization was due to the higher AMPK activation as well. Hence, it is tempting to speculate that during hyperglycemia, the EMD008 effects were at least in part due to AMPK activation. This reasoning agrees with previous work: *In vitro*, AMPK activation by HO-1 [53] or metformin [54] attenuated complement-induced cytotoxicity and TNF-α-induced inflammation, respectively. In turn, activation of AMPK can stimulate HO-1 expression and thereby attenuate cytokine-mediated cell death [55]. *In vivo*, in endotoxic mice, the metformin-induced attenuation of the hyper-inflammatory response ultimately resulting in increased survival was at least in part due to AMPK activation [56].

The present data link AMPK-activation with HO-1 induction and improved mitochondrial respiration under hyperglycemia. The latter implies improved oxidative glucose utilization, and as a consequence, an improved flow and removal of glycolytic metabolites. Increased levels of glycolytic metabolites are the primary cause for glucotoxicity [57], and their increased oxidative disposal should lead to lower metabolite levels and explain the observed tendency of a reduced glucotoxity. More importantly, if one considers that net glucose uptake is by part driven by an intracellular/extracellular concentration gradient, then lower intracellular metabolite levels should lead to a higher glucose uptake. To a certain extent, improved mitochondrial activity pulls extracellular glucose down the glycolytic pathway. Such a mechanism can explain the link between glucose uptake and AMPK stimulation and allows an increased glucose uptake without increasing intracellular metabolite levels and glucotoxicity. Improved hepatic mitochondrial respiration should improve the energy state. Various steps of gluconeogenesis require ATP, and it may well be that under septic conditions the gluconeogenic rate, albeit high, is limited by energy supply and the improved energy supply supersedes other effects of AMPK or EMD008 that downregulate gluconeogenesis under energy-sufficient conditions.

### Limitations of the study

All animals received continuous i.v. noradrenaline to achieve hemodynamic targets, but the noradrenaline infusion rate was significantly reduced by EMD008 during normoglycemia. It is unlikely, however, that the improved mitochondrial respiration under these conditions was only due to the reduced catecholamine administration: under hyperglycemic conditions, EMD008 also improved mitochondrial respiratory capacity despite virtually identical noradrenaline requirements. It could be argued that the higher rate of gluconeogenesis associated with the EMD008 treatment is in contrast to the reduced glucose formation expected from the actions of metformin. However, our finding is in line with previous observations in this model demonstrating that higher rates of hepatic gluconeogenesis coinciding with unchanged or even lower catecholamine infusion rates indicate improved hepatic metabolic performance [27, 58].

## Conclusions

During resuscitated, polymicrobial, murine septic shock, glycemic control either by reducing glucose infusion rates or by using the newly developed anti-diabetic drug EMD008 improved glucose uptake and thereby both liver tissue oxidative phosphorylation and maximal O_2_ uptake. The EMD008 effects were more pronounced during hyperglycemia and coincided with attenuated markers of tissue apoptosis. These beneficial effects of glucose control were at least in part due to up-regulation of HO-1 and activation of AMPK.

## Electronic supplementary material

Additional file 1: **Details of the modeling approach to analyze the glucose isotope data.** (PDF 260 KB)
