# Supplementary material for: Effects of glycemic control on glucose utilization and mitochondrial respiration during resuscitated murine septic shock
Source: Intensive Care Med Exp. 2014 Jun 3;2:19. doi: 10.1186/2197-425X-2-19 (PMC4678133; doi:10.1186/2197-425X-2-19)
Supplement: Supplementary file 1 — Additional file 1: Details of the modeling approach to analyze the glucose isotope data. (PDF 260 KB) [file 40635_2014_5020_MOESM1_ESM.pdf]

Supplement
Effect of glycemic control on glucose utilization and mitochondrial respiration during resusciated murine septic shock

Josef A. Vogt[1], Ulrich Wachter[1], Katja Wagner[1], Enrico Calzia[1], Michael Gröger[1], Sandra Weber[1], Bettina Stahl[1], Michael Georgieff[1], Pierre Asfar[2], Eric Fontaine[3], Peter Radermacher[1], Xavier M. Leverve[3], and Florian Wagner[1]

[1]Sektion Anästhesiologische Pathophysiologie und Verfahrensentwicklung, Klinik für Anästhesiologie, Universitätsklinikum, Ulm
[2]Département de Réanimation Médicale et de Médecine Hyperbare, Centre Hospitalier Universitaire, and Laboratoire de Biologie Neurovasculaire et Mitochondriale Intégrée, CNRS UMR 6214 - INSERM U1083, PRES L'UNAM, Angers, France
[3]Laboratoire de Bioénergétique Fondamentale et Appliquée, Université Joseph Fourier, Grenoble, France

Abstract

Details are presented of a modeling approach to evaluate ^{13}C-glucose tracer data. The model expresses glucose uptake and gluconeogenesis as functions of two controlling variables, namely plasma glucose concentration and hepatic AMPK activation.

Modeling approach

In the follwing we derive formulas to estimate gluconeogenesis and glucose uptake from plasma tracer enrichments obtained from a constant infusion of a mixture of unlabeled and $^{13}C_6$ labeled glucose assuming metabolic steady state conditions. The analysis of the resulting tracer enrichment in plasma is based on the metabolic structure shown in fig 1. The infused tracer is diluted by endogenous glucose production, whose rate is denoted as GNG (*gluconeogenesis*). Let x^* be the amount of labeled and x^0 that of unlabeled glucose in plasma. For the mole fraction of labeled material one obtains:

$$m_x^* = \frac{x^*}{x^* + x^0};$$

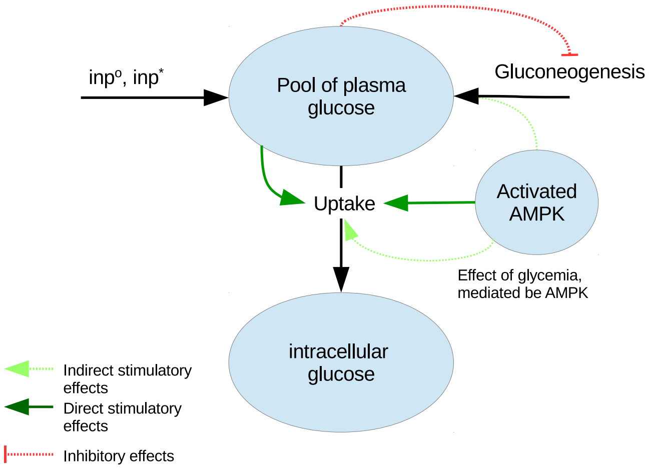

Figure 1: **structure of the model used to evaluate ^{13}C-Tracer data:** A one pool model is assumed, with the plasma glucose pool as central, whole body compartment. Gluconeogenesis feeds into this pool, and glucose uptake starts from it. Glucose uptake is controlled by glucose concentration and AMPK activation. In a simplified version, AMPK activation is triggered by glycemic effects, and hence glycemia exhibits a direct and an indirect, AMPK mediated effect. inp^o, inp*: Exogenous input of unlabeled (inp^o) and labeled (inp*) glucose.

With inp^* as the infusion rate of the labeled glucose the rate of gluconeogenesis can be estimated from the mole fraction of the labelled plasma glucose[1] as:

$$GNG = \frac{inp^*}{m_x^*}; \tag{1}$$

Based on model structure shown in fig 1 the combined glucose input and GNG matches its whole body uptake and the following balance holds:

$$\text{uptake} = GNG + inp_0 + inp^*; \tag{2}$$

To relate GNG and uptake values, estimated from tracer data to measurements of glucose concentration (x) and AMPK stimulation(z) the GNG and glucose uptake rates are expressed as a linear functions:

$$\text{uptake} = k_1 + k_2 x + k_3 z; \qquad GNG = k_4 + k_5 x + k_6 z; \tag{3}$$

With the proposed linearity the balances above also hold for the mean values of an experimental group and one can subtract the eqns 3 applied on group mean values from the same eqns, applied on a actual measurement set to obtain balances pertaining to 'centered values' as:

$$\Delta GNG + \Delta inp^0 + \Delta inp^* = k_2 \Delta x + k_3 \Delta z \tag{4}$$

$$\Delta GNG = k_5 \Delta x + k_6 \Delta z \tag{5}$$

Where Δ denotes the difference between actual value and group mean value. Measureable quantities are ΔGNG,Δx, Δz, the input values are known from

the experimental design. In the ideal case, there should be a set of coefficients k_2, k_3, k_5 and k_6 for which the measured quantities (ΔGNG, Δx and Δz), satisfy the eqns 4 and 5 for each data set, after correction for measurement errors. Hence, a set of coefficients is searched, for which the corrected values satify eqns 4 and 5 and were these corrected values came as close a possible to measured values. This dual task is performed using an orthogonal regression. The regression used corresponds to standard orthogonal regression, that was extended such that the variables for concentration and flow rates as well as the the corrected values for glucose concentration and AMPK activation have to satisfy eqns 4 and 5. The approach was implemented with the bayesian statistical package *stan* [2], which can be considered be a further development of the established WINBUGS/BUGS program[3], the orthogonal regression was extended based on the example demonstrated in[4], chapter 14.1. The corresponding source-script is shown in the section 'Source code' below. In a first analysis the data were evaluated using a separate set of coefficients for the vehicle and imeglimin group. The first two columns of table 1 show the corresponding determined coefficients. There is a collinearity between concentration and AMPK measurements which precludes determining the coefficients pertaining to AMPK for a separate analysis for each group. Hence, the coefficients for AMPK were set to zero for a separate analyse. Figure 2a shows the resulting uptake curve predicted for different concentration values. When the same set of coefficients was applied simultaneously for the vehicle and the imeglimin group an impact of AMPK could be separated from a concentration effect. A functional dependency on two variables is difficult to display. However, in average there is a positive correlation between AMPK activation and glucose concentration for the imeglimin group and a negative correlation for the vehicle groups. This allows to approximate the AMPK activation as a function of the plasma glucose concentration as:

$$\Delta z = p_0 + p_1 \Delta x. \tag{6}$$

The coefficients p_0, p_1 were determined from the group mean values for AMPK activation and glucose concentration. Different coefficients were obtainef for the imeglimin and vehicle group. Inserting the approximated AMPK values into eqn 4, and using the coefficients, shown in the third column of table 1 gives separate relations to express the uptake as a direct function of the concentration and an indirect concentration effect, mediated by AMPK. As there were different AMPK/(glucose concentration) relations for the vehicle and imiglimin group, different curves were obtained for the two different groups. They are shown in fig 2b in the main body of the paper.

source code

The code below demonstrates a separate analysis of the imeglimin and vehicle group. For the implementation eqns 4 and 5 are combined and solved for the glucose concentration:

$$k_5 \Delta x + k_6 \Delta z + inp_0 + inp^* = k_2 \Delta x + k_3 \Delta z - \tag{7}$$

$$\Delta x = \frac{(k_6 - k_3)\Delta z + inp_0 + inp^*}{k_2 - k_5} \tag{8}$$

Table 1: glucose uptake and GNG as a function of plasma glucose concentration and AMPK stimulation, based on eqn. 3

coefficient[c]	separated determination Imeglimin	vehicle	combined
	mean (95% confidence range)		
uptake coefficients			
k_1	-1.0(-2.4:-0.1)	-1.9(-6.6:-0.4)	-2.5(-3.9:-1.6)
k_2 (*100)	**2.4**(1.7:3.5)[a]	**2.4**(1.7:4.2)	**2.50**(2.0:3.2)
k_3	0.12(0:0.92)	0.29(0:2.00)	**1.6**(0.6:2.7)
GNG coefficients			
k_4	**0.87**(0.69:1.14)	**0.74**(0.58:0.92)	**0.82**(0.69:0.97)
k_5 (*100)	**-0.38**(-0.61:-0.22)[a]	**-0.29**(-0.42:-0.17)	**-0.34**(-0.46:-0.24)

Bold numbers: statistically different from zero. [a] statistically equivalent with corresponding determination for the vehicle group. [c] Units for the coefficients are mg/dl/min (k_2, k_4) or 1/min otherwise.

The following code holds for the case that $k_6 = 0$, or no impact of AMPK activation on gluconeogenesis. The code is used with the Stan package[2] that implements a Monte-Carlo, Marcov-Chain sampling algorithm, which in essence draws a series of random samples from the probability, that a set of flux rates and coefficients reflects the true values. The section 'model' defines the random processes and their interrelations, which are used to define the probability for flux rates and coefficients, given the measured values. The section 'generated quantities' calculates for the 'random draws' of coefficients uptake values as a function of concentration values. 10000 samples were drawn in this way and mean and quartile values were assessed from these 10000 samples.

```
data {          //definition of input data
int<lower=0> Na;    // number of cases for imeglimin group
int<lower=0> Nb; // number of cases for vehicle group
int<lower=0> N; // total number of cases
// suffix a or _a refers to imeglimin group
// suffix b or _b refers to vehicle group
real gnga[Na];
real conca[Na];
real ampka[Na];
real tracerInputa[Na];
real traceeInputa[Na];
... comparable entries for vehicle group
}

transformed data {
real inpCa[Na];
real gngCa[Na];
real concCa[Na];
real ampkCa[Na];
real ampkMa;
real inpMa;
real concMa;
```

```
real gngMa;
... comparable entries for vehicle group
real nu;
real<lower=0> alpha_conc;
real<lower=0> beta_conc;
real<lower=0> alpha_gng;
real<lower=0> beta_gng;
real sigma_ampk;

// calculation of total glucose input
for (i in 1:Na) {
  inpCa[i]<-tracerInputa[i]+traceeInputa[i];
  }
 for (i in 1:Nb) {
     inpCb[i]<-tracerInputb[i]+traceeInputb[i];
  }
ampkMa <- mean(ampka);
inpMa <- mean(inpCa);
concMa <- mean(conca);
gngMa <- mean(gnga);
ampkMb <- mean(ampkb);
inpMb <- mean(inpCb);
concMb <- mean(concb);
gngMb <- mean(gngb);
// measurement values a centered, or expressed as offset from mean values
  for (i in 1:Na) {
  inpCa[i]<-inpCa[i]-inpMa;
  gngCa[i]  <- gnga[i]-gngMa;
  concCa[i] <- (conca[i]-concMa)*0.01;
// centration is scaled to be in the same range as AMPK activation
  ampkCa[i] <- ampka[i]-ampkMa;
  }
... comparable entries for vehicle group
// vague prior for measurement errors of gluc. concentration and
// gluconeogenesis
alpha_conc <- 300.0;
beta_conc <- 2.0;
alpha_gng <- 350.0;
beta_gng <- 0.5;
nu<- 14.0;
// degree of freedom for the student-t distributions, which are used to
// describe random measurement errors.
// a student-t distribution is used to account for tailing.
sigma_ampk<-0.022;
//experimentally defined standard deviation of AMPK measurements
}

parameters {
  real k2_a;
  real p3a;
```

```
  real k5_a;
  real k2_b;
  real p3b;
  real k5_b;
  real<lower=0> tau_gng;
  real<lower=0> tau_conc;
  real ampk_model_a[Na];
  real ampk_model_b[Nb];
}

transformed parameters {
real<lower=0> sigma_conc;
real<lower=0> sigma_gng;
real<lower=0> k3_a;
real<lower=0> k3_b;
sigma_conc <- pow(tau_conc,-0.5);
sigma_gng <- pow(tau_gng,-0.5);
k3_a<-exp(p3a);  // parameter k3 is assumed to be log-normal distributed,
//  allowing a tailing towards higher values
k3_b<-exp(p3b);
}

model {
// this section describes the model equations
// used to assess the various distributions
   tau_conc ~ gamma(alpha_conc,beta_conc);
    // prior for the precision of concentration measurements
   tau_gng ~ gamma(alpha_gng,beta_gng);;
    // prior for the precision of GNG determinations
   k2_a ~ normal(2,5);
   p3a ~ normal(1,5);
   k5_a ~ normal(0,1);
   for (n in 1:Na){
       real h;
       real c1;
       // model value for ampk calculated as described in
       //  chapter 14.1 in the stan-manual,  here a
       //  student t distribution is used for more robust determinations
       ampk_model_a[n] ~ student_t(ampkCa[n],sigma_ampk);
       h <- inpCa[n] -k3_a*ampk_model_a[n];
       // calculation of gluc. concentration using eqn. 7
       c1 <- h/(k2_a-k5_a);
       concCa[n] ~ student_t(nu,c1,sigma_conc);
       gngCa[n] ~ student_t(nu,k5_a * c1, sigma_gng);
  };
... comparable entries for vehicle group
}

generated quantities {
// this section defines variables and corresponding formulas
```

```
// to assess posterior distributions
real sseConca;
real sseGnga;
real sseAmpka;
real sseConcb;
real sseGngb;
real sseAmpkb;
real k1a;
real k1b;
real predFluxA[41];
real predFluxB[41];

sseConca <- 0.0;
sseGnga <- 0.0;
sseAmpka <- 0.0;
for (n in 1:Na){
// calculation of sum of square errors (sse...)
// as a measure of fit-quality
  real h;
  real c1;
  real gng1;
  real delta;
  h <- inpCa[n]-k3_a*ampk_model_a[n];
  c1 <- h/(k2_a-k5_a);
  delta <- (c1-concCa[n])/sigma_conc;
  sseConca <-sseConca + delta*delta;
  gng1<- k5_a * c1;
  delta <- (gng1-gngCa[n])/sigma_gng;
  sseGnga <-sseGnga + delta*delta;

  delta <- (ampk_model_a[n]-ampkCa[n])/sigma_ampk;
  sseAmpka <-sseAmpka + delta*delta;
}
... comparable entries for vehicle group

 k1_a<-inpMa+gngMa-k2_a*concMa*0.01-k3_a*ampkMa;
 k1_b<-inpMb+gngMb-k2_b*concMb*0.01-k3_b*ampkMb;
// prediction for uptake, as shown in figur 2a
 for (n in 1:41){
   real cpred;
   cpred<-dConc+(n-20)*0.02;
   predFluxA[n]<-k1_a+k2_a*(cpred+1.1809)+k3_a*ampkMa;
   // cpred refers to centered values, and 1.1809 is the mean concen-
   // tration for the imeglimin group
   predFluxB[n]<-k1_b+k2_b*(cpred+1.3301)+k3_b*ampkMb;
   // 1.3301 is the mean concentration for the vehicle group
}
```

The use of a studend-t distribution in the context of a regression to account for a tailing , that exceed that of normal distribution is motivated by section 12.3

of the stan-manual [4].

References

[1] Vogt JA, Chapman TE, Wagner DA, Young VR, Burke JF. Determination of the isotope enrichment of one or a mixture of two stable labelled tracers of the same compound using the complete isotopomer distribution of an ion fragment; theory and application to in vivo human tracer studies. Biological mass spectrometry. 1993;22(10):600–612.

[2] Stan Development Team. Stan: A C++ Library for Probability and Sampling, Version 2.0; 2013. Available from: `http://mc-stan.org/`.

[3] Lunn DJ, Thomas A, Best N, Spiegelhalter D. WinBUGS - A Bayesian modelling framework: Concepts, structure, and extensibility. Statistics & Computing. 2000;10(4):325–337.

[4] Stan Development Team. Stan Modeling Language User's Guide and Reference Manual, Version 2.0; 2013. Available from: `http://mc-stan.org/`.
